# Supplementary material for: Expanding the MECP2 network using comparative genomics reveals potential therapeutic targets for Rett syndrome
Source: eLife. 2021 Aug 6;10:e67085. doi: 10.7554/eLife.67085 (PMC8346285; doi:10.7554/eLife.67085)
Supplement: Supplementary file 3. [file elife-67085-supp3.docx]

**Sequences of primers used for RT-PCR**

| **Gene** | **Sequence** |
| --- | --- |
| **IL-13** | S: CGAGAAGACCCAGAGGATG  AS: GTCTCGGACATGCAAGCTG |
| **TNF-α** | S: CCACGCTCTTCTGCCTGCT  AS: GCTTGTCACTCGGGGTTCG |
| **IL-1α** | S: CCAAGATGAAGACCAACCA  AS: GCCAAGCACACCCAGTAGT |
| **CD86** | **S:** TCAATGGGACTGCATATCTGCC  **AS:** GCCAAAATACTACCAGCTCACT |
| **CD206** | S: CATCAGGGTGCAAGGAAGG  AS: GTCCAGGCACTGAAAGTGGA |
| **βIIItubulin** | S: AGCGGATCAGCGTCTACTAC  AS: TCAGGCCTGAAGAGATGTCC |
| **GFAP** | S: TCCTTGACCTGCGACCTG  AS: TCTGCCCCTCTTCCTCCA |
| **BDNF** | S: CCCCCATGAAAGAAGCAAAC  AS: CGTGTTCGAAAGTGTCAGCC |
| **S12** | S: TGCTGGAGGTGTAATGGACG  AS: CAAGCACACAAAGATGGGCT |
